# The Long-Term Mental Health Consequences of Torture, Loss, and Insecurity: A Qualitative Study Among Survivors of Armed Conflict in the Dang District of Nepal

**DOI:** 10.3389/fpsyt.2019.00941

**Published:** 2020-01-15

**Authors:** Hanna Kienzler, Ram P. Sapkota

**Affiliations:** ^1^ Department of Global Health & Social Medicine, King’s College London, London, United Kingdom; ^2^ Department of Psychiatry & Douglas Mental Health University Institute (DMHUI), McGill University, Montreal, QC, Canada

**Keywords:** trauma, PTSD, idioms of distress, mental health, global mental health, violence, torture, Nepal

## Abstract

**Background and Objectives:** Nepal has witnessed several periods of organized violence since its beginnings as a sovereign nation. Most recently, during the decade-long Maoist Conflict (1996–2006), armed forces used excessive violence, including torture, resulting in deaths and disappearances. Moreover, there is widespread gender-, ethnic- and caste-based discrimination, and grossly unequal distribution of wealth in the country. While the immediate mental health effects of the conflict are well studied, less is known about the long-term effects of the conflict. This article sets out to explain how Nepalese survivors of violence perceive their wellbeing and mental health, search for help and construct their health care pathways and therapeutic itineraries. The aim is to provide a better understanding of local explanatory models and healthcare behaviors.

**Methods:** Semi-structured interviews were carried out with 25 people (14 men, 11 women) aged 30 to 65 in Dang district in 2013. To elicit illness narratives, a translated and culturally adapted version of the McGill Illness Narrative Interview (MINI) was used. Additionally, participants were interviewed about their war experiences and present-day economic and social situations. The transcripts were coded using deductive and inductive approaches and analyzed through thematic analysis.

**Results:** The study provides insight into temporal narratives of illness experience and explanatory modules. Symptoms were found to be widespread and varied, and were not solely attributed to violent experiences and memories, but also to everyday stressors related to survivors’ economic, social, and familial situations. In terms of help- and health-seeking behavior it was found that participants resorted to various coping strategies such as social activities, avoidance, withdrawal, and substance use. Many participants had received biomedical treatment for their psychosocial problems from doctors and specialists working in public and private sector clinics and hospitals as well as different forms of traditional healing.

**Conclusions:** These results shed light on the long-term impact of the Nepalese conflict on survivors of extreme violence, highlighting local explanatory models and help- and health-seeking behaviors. These findings inspire recommendations for the development of context specific and holistic psychosocial interventions focusing on well-being, social determinants of health, and human rights.

## Introduction

Nepal, although never colonized by any other country, has witnessed structural and organized violence since its unification as a sovereign nation in the mid-eighteenth century including, most recently, during the decade-long Maoist Conflict (1996–2006) (1). During the Maoist Conflict, armed forces used excessive violence, including torture, resulting in deaths and disappearances. Moreover, there continues to be widespread gender, ethnic, and caste-based discrimination, and grossly unequal distribution of wealth. While the immediate mental health effects of the conflict are well studied, this article provides insight into the ways Nepalese survivors perceive their mental health problems, seek help, and respond to mental health treatment in the long-term.

Understanding the long-term effects of armed conflict on people’s mental health and wellbeing is crucial for several reasons. On the one hand, the long-term recovery from wars is complex as communities undergo reconstruction and reorganization of social and political life and struggle to provide adequate health and social support to those suffering from physical and mental health problems (2, 3). In contrast, support for survivors is usually provided on a short-term basis in the form of humanitarian assistance, which now increasingly also includes the provision of psychiatric and psychosocial services to address trauma-related mental health problems (4–8). One reason for the provision of short-term mental health support in the face of long-term suffering might be the fact that there is little systematic research into the long-term consequences of war and violence on people’s mental health and wellbeing.

Research tends to be conducted in the immediate aftermath of conflict periods, and the few existing longitudinal studies have tended to focus on small sets of psychological symptoms or syndromes or on high-income country settings (9, 10). Nevertheless, findings related to the long-term mental health effects of war among Bosnian and Croatian civilians have highlighted that psychological symptoms and general psychological distress decrease over time, and that only a subsample of survivors continue to suffer from high levels of psychological symptoms due to distinct war experiences and contemporary stressors (9, 11). Similarly, a study focusing on mental health outcomes among Sri Lankan adolescents four years after the tsunami highlighted the interplay between traumatic experiences, social support networks, and ongoing daily stressors. It was found that daily stressors were transmitters of the impact of trauma on adolescents’ mental health problems and that social support moderated pathways from trauma to daily impairment. Consequently, it was recommended that interventions focus “not only on trauma processing but also on reducing current stress and improving social support” (12, p. 487).

These findings are in line with previous research that has highlighted the importance of investigating how war-related stressors cause and/or amplify existing daily or chronic stressors, and how such chronic stressors mediate the impact of war on mental health and psychosocial functioning (13). It has further been emphasized that such an integrated understanding should inform the development and implementation of holistic and context-specific interventions (2). Holism is crucial in that interventions which solely focus on healing the effects of war exposure have been shown to have limited impact as long as people face ongoing exposure to structural violence and daily stressors related to poverty, job insecurity, and housing, among others. Context-specificity is important as both war-related and chronic stressors differ across geographic, economic, cultural and sociopolitical settings as well as by age and gender (see also 14).

Even though it is now widely accepted that there exists a bidirectional relationship between war exposure and social determinants of health even in the long-term, the development of and evidence-base for holistic and culturally sensitive context-specific interventions continues to be sparse (15, 16). Johnson, (15) has linked the reasons for this gap to several factors including that (i) responses to social determinants are political rather than merely clinical; (ii) clinical professionals and researchers may feel as though they are overstepping boundaries when intervening in people’s private lives and may feel overwhelmed or even helpless in the face of social challenges; (iii) the range of targets for social interventions is wide as it encompasses national, communal, family and individual levels; (iv) there are other professions that are viewed as already intervening on social levels; and (v) the mental health field continues to be domineered by psychiatry and psychology with a less developed literature and work-force in social care. Despite these obstacles, calls for investing in national and local policies that directly target the underlying social causes of mental illness are becoming louder.

Our article seeks to contribute to this nascent field by exploring how Nepalese survivors of the Maoist Conflict (1996–2006) experience and reflect on their mental health and wellbeing, help- and health-seeking, as well as received treatment seven years after the end of the conflict. We begin by reviewing the epidemiological, public health and anthropological literature in order to establish mental health outcomes during and right after the conflict. This is followed by findings of our own investigation into temporal narratives of illness experience; salient prototypes regarding current health problems; and causal attributions and treatment expectations. In addition, help- and health-seeking behavior and pathways to care will be explored as they unfold in the present. The findings inspire recommendations for the development of holistic as well as culture- and context-specific, and long-term psychological and social interventions focusing on well-being, social determinants of health, and human rights.

## Background Literature Review

The Nepalese Civil War, also known as the Maoist Conflict or Maoist Revolution, was a decade-long armed conflict between the Communist Party (Maoists) and the government of Nepal that ended with a comprehensive peace agreement in 2006. Between 1996 and 2006, armed forces on both sides of the conflict are alleged to have used excessive violence resulting in deaths and enforced disappearances. The conflict has resulted in between 13,000 to 16,500 deaths, with thousands more missing. More than 150,000 people were internally displaced and more than 100,000 incidents of torture have been reported (17–19). Despite being considered one of the poorest countries in the world and affected by a long history of political instability and frequent natural disasters (i.e., seasonal floods, landslides and earthquakes), the country has achieved noteworthy progress in reducing poverty, getting children into school and improving maternal and child health. For example, the infant mortality rate has gone down from 108 per 1,000 live births in the1990s to 27.4 in 2017. Moreover, the percentage of people living below the national poverty line decreased to 21.6 in 2017 from 42 in 1990. According to the human development index (HDI) report, Nepal is now among the medium development countries with an HDI ranking of 149 out of 189 countries with a per capita income of US $1004, life expectancy of 70.6 years and adult literacy rate of 59.6% (20, 21).

While there have been rapid social, cultural, political, and economic changes in recent years, inequalities in wealth distribution and according to gender, caste, and ethnicity prevail. Theoretically, Nepal abolished caste-based discrimination in 1963; however, caste-based exclusion is still widespread and affects every aspect of life, particularly among Dalits (“untouchables”) in rural areas of Nepal (22, 23). Moreover, there is a high rate of domestic and other forms of gender-based violence (24). Thousands of women and girls are trafficked to India, the Middle East, and other countries for forced sexual exploitation, forced labor, and forced marriages every year (25). In fact, Nepal has one of the highest rates of child marriage in Asia (37% of girls are married before the age of 18) (26). These inequalities persist despite the fact that the Maoist Conflict is believed to have emerged from and sought to change extant poverty, unequal division of wealth, and ethnic, regional, and caste discrimination in the country (1, 27–29).

### The Effects of the Maoist Conflict on Mental Health

Mass violence during the Maoist Conflict as well as protracted and organized political violence and injustice have been shown to negatively affect the mental health of exposed populations (29, 30). High rates of psychiatric morbidity and disability were found among ethnically Nepali Bhutanese refugees (1, 31, 32), female survivors of human trafficking (25, 33), torture survivors (1, 19, 32, 34), and internally displaced persons (30, 35). Despite the lack of a Nepalese term equivalent to “trauma” and the coexistence of different local idioms of distress (36, 37), studies conducted in Nepal found high rates of symptoms consistent with PTSD. A case control survey comparing tortured and non-tortured Bhutanese refugees living in Nepal found a higher prevalence of PTSD (14 vs. 3%), anxiety (43 vs. 34%) and depression (25 vs. 14%) among those who had experienced torture (32). Similar findings were reported from another case-control study of PTSD among tortured (43%) and non-tortured (4%) Bhutanese refugees in Nepal (38). Studies have also revealed a higher prevalence of psychosocial and mental health problems, including PTSD, among displaced persons relative to the general population. A cross-sectional study conducted during the armed conflict shows that 53% of displaced persons suffered from PTSD, while 81 and 80% suffered from anxiety or depression, respectively (35). In 2008, the Center for Victims of Torture (CVICT) and the Transcultural Psychosocial Organization (TPO) of Nepal, carried out an epidemiological survey with 720 randomly selected adult samples in three districts of Nepal. It was found that 28, 23, and 10% of the sample met the symptom criteria for depression, anxiety and PTSD respectively (39). The differences in prevalence rates between these various studies has been considered difficult to explain and is most likely due to methods and sampling procedures (19).

Besides trauma-related mental disorders, research has also been conducted on local expressions of distress and Nepali “ethnopsychology” (37, 40). Kohrt and Harper (41) indicate that there are five dimensions of self-connected to mental health in Nepal, including what is called the *man* (heart–mind); *dimāg* (brain–mind); *jiu* (physical body); *sāto* (spirit); and *ijjat* (social status). The *man* and *dimāg* are particularly relevant for mental health: too much activity in the *man* is associated with psychosocial complaints while *dimāg* dysfunction is associated with being crazy, mad, or psychotic. Other studies have identified local idioms of distress and found that *jham-jham* (numbness and tingling) and *gyastrik* (dyspepsia) are common complaints that can be associated with depression (42). Among Nepali speaking Bhutanese refugees in Nepal, Sharma and Van Ommeren (18) have listed a number of idioms of distress including *dukha lāgyo* (sadness), *dar laagyo* (fear), *jharko lāgyo* (irritation), *jiu sukera gayo* (drying of the body) and *kat kat khancha* (tingling and burning sensation). Kohrt and Hruschka (37) have conducted a careful study inquiring into Nepali idioms of distress in connection to conceptions of local ethnopsychology and ethnophysiology. The study highlights that terms such as “depression” and “trauma” are not widely understood while local expressions such as *dukha* (trouble, grief, sorrow, misfortune, and hardship), *chinta* (anxiety, worry, trouble), *dar* (fear, dread, awe, terror), or *ris* (anger, wrath, ill temper, resentment) are commonly used to refer to related emotional states. Other studies have drawn attention to high rates of somatization among survivors of violence (38, 43).

Taken together, these studies make apparent that psychological trauma is a multifaceted concept and that people in Nepal express experiences of psychological trauma through a variety of idioms reflecting impacts on the heart-mind, brain-mind, body, spirit and social status as well as differences in perceived types of traumatic events, symptom sets, emotion clusters, and vulnerability. Less research is available on the long-term impact of the Maoist Conflict on mental health; the ways in which these long-term effects intersect and interact with chronic daily stressors; and what locally meaningful and appropriate mental health interventions and social support mechanisms could or should look like (an important exception is the development of ecological interventions for former child soldiers in the armed conflict (44, 45).

### Access to Mental Health Treatment and Care in Nepal

In Nepal, available treatment for mental health problems is pluralistic. Among commonly practiced types of healthcare are Ayurveda, Tibetan medicine, and homeopathy, which are mostly available in cities, and widely available traditional healing (ritual or faith-based healing, astrology, herbal medicine) and biomedicine (42, 46, 47). Interestingly, it has been found that biomedical treatment related to mental health is sought only as a last resort by most people—generally after all other means of healing and treatments fail (42, 48, 49). This might be related to the scarcity of mental health professionals and service availability outside the boundaries of major cities (50). It has been estimated that there are only about 110 psychiatrists, 15 clinical psychologists, and 400–500 paraprofessional psychosocial counsellors in the entire country (50, 51). In contrast, traditional healers are readily accessible with approximately one traditional healer per 650 persons (41). Other reasons proposed for the low uptake of biomedical practice include Nepali cultural beliefs attributing mental health problems to spirit afflictions (48) and the lack of recognition of mental health problems except for “madness” in Nepali ethnopsychology and associated stigma related to mental illness (36, 41).

Despite the apparent limitations of biomedical and specialized mental health care, it has been noted that the western biomedical model of psychiatric care is being increasingly recognized and practiced, especially in hospitals and private clinics in major cities and by non-governmental organizations (50, 52–54). In collaboration with NGOs, the Nepal government is in fact working on developing a district-level mental health plan in an effort to integrate mental health care into primary health care and to revise mental health policy. There has also been a burgeoning interest in understanding local cultural knowledge of mental illness and care, and translating that knowledge into culturally appropriate interventions (36, 55). However, due to a weak governing body, these efforts have not been translated into sustainable mental health care strategies.

## Methodology

This research builds on previously conducted epidemiological research identifying prevalence rates of mental health problems, factors associated with poor mental health and protective and risk factors in post-conflict Nepal (39). Based on this work, a clinical trial was carried out testing a novel treatment aiming to diminish traumatic memory and its effects among Nepalese torture survivors suffering from chronic PTSD. The means selected to accomplish this was the implementation of a small, pilot randomized controlled trial (RCT). Our main hypothesis tested the efficacy of reconsolidation blockade using the beta-blocker propranolol vis-à-vis the selective serotonin reuptake inhibitors (SSRI) paroxetine, the “gold standard” pharmacological treatment for PTSD across the world. In this study, reconsolidation blockade using propranolol was as effective as paroxetine for treating PTSD in a sample of Nepali torture survivors (publication forthcoming). The results presented in this article form part of the qualitative component of the research project which was complementary and explored the ways in which people suffering from trauma-related health problems as well as ongoing socioeconomic stressors perceive their mental health problems, seek help and construct their health care pathways and therapeutic itineraries.

### Setting

This qualitative study took place in Nepal’s Dang district from mid-February until April 2013. Dang district is located approximately 280 km west of Kathmandu. The district’s population is estimated to be 552,583 people belonging to multiple linguistic and ethnic groups including Tharu (30%), Chhetri (25%), Magar (14%), Brahman-Hill (10%), Kami (6%), and other (16%). Dang was badly affected during the Maoist armed conflict. Civilians were subjected to extreme violence including killing, abduction, disability and injury due to landmines, torture and sexual abuse. The region continues to be affected by poverty, deprivation and discrimination (https://nepalmap.org/profiles/district-60-dang/).

Poverty and other social determinants have a decidedly negative impact on people’s health and wellbeing. Approximately 39.3% of children under the age of five are malnourished, only 55.3% of the population have access to safe drinking water, and access to healthcare is limited with only one hospital, four primary health centers, ten health posts and 26 sub-health posts available in the region (see https://nepalmap.org/profiles/district-60-dang/). A recently conducted epidemiological study that involved randomly selected participants from three districts identified that living in Dang was a risk factor for psychosocial and mental health problems (39). Yet despite the fact that psychosocial wellbeing and mental health are increasingly recognized by the government to be important issues related to the overall health status of the population, no working care system exists to address the needs of people with mental health problems.

### Study Population and Sampling

Study participants were adult male and female survivors of torture and violence during the Maoist Conflict. They were purposively recruited by combining criterion and snowball sampling approaches. Criteria for inclusion were being over the age of 18 years, having been directly affected by the Maoist Conflict, and being a resident of the Dang district. Local research assistants who had been hired as case finders for the clinical trial component of this study helped to identify potential participants for the qualitative component of the project. Potential participants were then visited at their homes and screened to identify whether their traumatic experiences dated back to the armed conflict. If they met all inclusion criteria, they were recruited to the study. Additionally, a snowball sampling approach was followed whereby each participant was asked if they knew of any person who had been affected by the conflict like themselves and if they could put us in touch with them for the purpose of screening and recruitment.

The sample consisted of 25 participants (14 men, 11 women) aged between 30 and 65. All of them were civilians with three former Maoist fighters (2 male, 1 female) among them. Most lived in villages while five participates lived in towns. Most of the participants were farmers and very few held additional occupations such as health volunteer (n = 1), teacher (n = 1), VDC leader (n = 2), and restaurant owner (n = 1). The majority had some reading and writing skills, with three participants being illiterate. Most participants considered their economic situation “simple” but sufficient to make a living and support their families. Thirty-two percent of the interviewees described themselves as poor and experienced difficulty providing for their families, particularly their children, in terms of food, clothing and school supplies (See **Table 1**).

**Table 1:** Study participants including traumatic experiences and most important daily stressors.

Interview	Gender	Age	Occupation	Traumatic event	Most important daily stressors
Interview 1	F (widow)	44	Farmer	Son shot and killed	Economic difficulties Worries about how to provide for children Widowhood Social exclusion Lack of family support
Interview 2	F	Almost 50	Farmer	Killing of daughter	Inability to visit grave of daughter Longing for daughter
Interview 3	M	Middle aged^*^	Farmer	Torture	Economic difficulties Lack of support from the government Disability Social exclusion
Interview 4	F	Older	Framer	Violence and threats against family	Worries Missing children who are working abroad
Interview 5	M	Older	Farmer	Torture Almost drowned	Worries Missing children who are working abroad
Interview 6	F (widow)	Middle aged	Farmer	Killing of husband Flight	Widowhood Social exclusion Lack of family support
Interview 7	F	40	Farmer Retired Maoist fighter	Witnessing violence	Economic difficulties Social exclusion Worries about how to provide for child Missing family who live far
Interview 8	M	32	Farmer Retired Maoist fighter	Torture	Economic difficulties Disability Social exclusion Worries about how to provide for child
Interview 9	M	45	Farmer	Torture	Economic difficulties
Interview 10	F (widow)	38	Health volunteer	Loss of family members Suicide of a family member	Widowhood Social exclusion Lack of support from government
Interview 11	M	Older	Former VDC leader Farmer	Abduction	Lack of support from government
Interview 12	M	Older	Former VDC leader Farmer	Torture	Economic difficulties
Interview 13	F (widow)	36	Unemployed/ working at home	Killing of husband	Widowhood Social exclusion Economic difficulty Unemployment
Interview 14	M	Middle-aged	Teacher	Torture	Economic difficulties
Interview 15	M	75	Farmer	Killing of son	Worries
Interview 16	M	41	Farmer	Torture Imprisonment Kidnapping	Economic difficulties
Interview 17	M	30	Restaurant owner Retired Maoist fighter	Imprisonment Torture	Lack of family support
Interview 18	M	63	Farmer	Displacement Looting Torture	Social exclusion Lack of support from government
Interview 19	F	46	Farmer	Torture Witnessing torture	Economic difficulties
Interview 20	F	Older	Farmer	Killing of son	Worries
Interview 21	M	63	Farmer	Killing of 3 sons	Lack of family support Economic difficulties
Interview 22	F	55	Farmer	Killing of 3 sons	Economic difficulties
Interview 23	M	Older	VDC representative	Witnessing violence	Community responsibilities Lack of support from government
Interview 24	F (widow)	45	Unemployed	Killing of husband	Widowhood Social exclusion Economic difficulties Unemployment
Interview 25	M	Middle-aged	Farmer	Abduction of mother	Lack of support from government

*Many study participants did not know how old they were.

### Data Collection and Analysis

Data were collected through semi-structured interviews and observations carried out during and after interviews of interactions between household members and living standards. The topic guide included questions about family structure, socioeconomic situation, and work, and inquired into experiences during the Maoist Conflict. To elicit illness narratives, we used a translated and culturally adapted version of the McGill Illness Narrative Interview (MINI; Nepalese translation: https://www.mcgill.ca/tcpsych/files/tcpsych/mini-nepali-revised_v.3.1_0.pdf) (56). The MINI is a theoretically driven, semi-structured, qualitative interview protocol which is divided into three sections: i) a basic temporal narrative of symptom and illness experience; ii) salient prototypes related to current health problems, based on the previous experience of the interviewee, family members or friends and mass media or other popular representations^1^; and iii) any explanatory models, including labels, causal attributions, expectations for treatment, course and outcome. Additionally, we used the supplementary sections of the MINI which explore help-seeking and pathways to care, treatment experience, adherence and impact of the illness on identity, self-perception and relationships with others.

Interviews were conducted with individual participants except in two instances where couples were interviewed together based on their specific request for such an arrangement. Interviews were carried out by both authors. They were conducted in Nepali by the second author and involved, when the first author was interviewing, the help of a Nepali translator. The interviews lasted between 40 min and 1 h and were, with the consent of study participants tape-recorded and later transcribed verbatim and translated into English. Both authors read the transcripts carefully before coding them by combining deductive and inductive approaches (57, 58). The data were analyzed using thematic analysis (59). Specifically, the coded data were, first, categorized and linked by relationship into categories. Thereupon, links were established between the categories and defined properties such as phenomena, causal conditions, context, action strategies and consequences. Through an interpretative process, it was possible to identify the core themes which are presented in the following results section.

### Ethics Approval

Ethical approval for this study was received from the Research Ethics Board of the Douglas Mental Health University Institute in Montreal, Canada and from the ad-hoc ethical committee at the Department of Psychiatry and Mental Health at Tribhuvan University Teaching Hospital in Kathmandu, Nepal. We obtained written informed consent for the publication of the data from the participants.

## Results

### Narratives of Symptoms and Illness Experiences

To develop an in-depth understanding of how survivors of the armed conflict in Nepal perceive their distress and related health problems seven years after the conflict, we elicited basic temporal narratives of symptoms and illness experiences. We asked the following questions: (i) From what kind of emotional or physical health problems do you suffer? And, since when do you suffer from these health problems? (ii) When you have these health problems, what kinds of symptoms do you experience? (iii) What else happens when you experience these (symptoms)? These questions were designed to act as a prompt for further discussion concerning the frequency, intensity and location of symptoms.

Respondents listed many and a wide range of symptoms of emotional, physical, and psychosomatic nature. The following excerpt of an interview with an older couple who had been tortured, beaten, and almost drowned to death by Maoist fighters is exemplary:

Husband: In my dreams I see people dying and (I feel) dizzy and shaking, I see those same *Maobadi* (Maoists). I see the same unfortunate event in my dreams.

Q: Does this happen to you (wife) too? Seeing something like that in your dreams?

Wife: Yes, the same thing happens to me too. Sometimes (I dream) that I’m walking along the river, and sometimes that they’ve thrown me into the river. (…) When I’m about to wake up and my heart is racing, I remember that event and I feel that maybe it is still happening? Maybe they’ll come back and try to kill my husband again? Maybe they’ll kill me?

Q: When those kinds of things come to your mind, do you experience any emotional or physical symptoms? If so, what kind of symptoms do you experience?

Wife: In my body unusual things happen, my stomach hurts, my mind is restless (*man ātinchha*), sometimes my chest hurts, my heart hurts (*mutu dukhchha*); during this time, it feels like my ribs hurt, my finger doesn’t function well because my nerve is swollen so I cannot even knead dough, I can’t cut grass, you know I can’t even serve dinner well.

The couple refers to a combination of symptoms related to their gruesome experiences. In this short excerpt they mention nightmares and sleeping problems, dizziness and body shaking, pain in stomach, chest, ribs and heart, restlessness of the mind and swollen nerves. Frequently mentioned symptoms and feelings expressed by other study participants included sleeping problems (n=14), worries (n = 11), headache (n = 8), pain (n = 7), eating problems (n = 6), goosebumps (n = 6), anger (n = 5), fear (n = 5), emotional pain (n = 4), listlessness (n = 4), restlessness (n = 4), nightmares (n = 4), and mental tension (n = 4). Less frequently mentioned yet shared were back pain, weakness, stomach pain, thinking too much, heat in their bodies, and flashbacks. Other symptoms included heart pain, heart racing, chest pain, forgetting, stress, sweating, speaking problems, avoidance, hurting eyes, dizziness, constriction of the throat, high blood pressure, and vision problems. Finally, a number of symptoms were only mentioned by individual respondents (see footnote).^2^

Since study participants were allowed to freely list their symptoms, a wide range were mentioned which is in line with previous studies (see 18, 19, 35). While all respondents mentioned various symptoms (ranging from 2 to 19 per person), the illness narratives revealed that symptoms were not equally distributed. Men experienced slightly fewer symptoms than women (average number of symptoms for men 7.8 and for women 9.4), and among women, widows experienced more symptoms than non-widowed women (average symptoms for widows 11 and for non-widowed women 8). These discrepancies did not appear to be solely a reflection of the type or frequency of violence experienced during the conflict, but also of current social determinants, including participants’ economic situation, gender-based discrimination, and issues of marginalization and community exclusion (see below).

### Causal Attribution: Traumatic Experiences

Most participants stated that their symptoms had been initially caused by the violence and insecurity they had to endure and witness during the decade-long armed conflict. They reported that their symptoms increased or tended to reappear when they recalled the armed conflict and related violence, were confronted with persons who had inflicted violence on them or their family members, or visited places that reminded them of particular gruesome events.

Many of the participants listed a number of violent experiences including the loss of family members (n = 9), living through and surviving torture (n = 9), experiencing and witnessing kidnappings (n = 3), imprisonment (n = 2), and long-term displacement (n = 2). The loss of family members was experienced as extremely painful, affecting a person’s entire being emotionally, physically, and socially. A man told us about the shock he had felt when his son was shot in their family garden and how this experience haunts him until this very day. He said, “even now, my mind doesn’t work. If I think something, I forget about it immediately.” He continued to feel depressed, felt as though there was no one he could rely on, and had lost his faith in the gods: “I feel that the fasting (*barta*) for the gods and goddesses is useless. I don’t believe in any gods now.”

A woman highlighted the pain she experienced remembering how her daughter was murdered. Due to the fact that her daughter’s bodily remains were never located, she was still unable to find emotional closure. She stated, “Perhaps if I had seen my daughter’s corpse, if I had gotten the chance to cremate [her body], then maybe there wouldn’t be so much pain, but I didn’t get to see her corpse. It is unknown where they killed her or where they threw her body, and even now I feel maybe she’s still alive.” She explained that the grief and uncertainty caused her sleeping problems and unstable health.

My 14-year-old daughter died, the enemy killed her. The difficulty during that time was indescribable, I didn’t feel like eating, my health was not stable, no matter what (…). I withstood a lot of [emotional] pain. (…) It was difficult for me to even feed and dress myself. I didn’t get pennies from anywhere, I had difficulties to educate my children.

She went on to explain that remembering her daughter presently led to her feeling emotionally low which became particularly strong when she saw young girls passing by who were her daughter’s age as they made her wonder who her daughter would have become had her life not been cut short.

Mostly male participants reported that their suffering and emotional pain was caused by experiences of torture during the conflict. They reported gruesome mistreatment at the hands of government forces and Maoists including severe beatings with rifles, stones, and boots; breaking of bones; cutting; attempts at drowning; blind folding; and shackling with ropes and sticks. A village teacher provided the following testimony:

It was around 10 o’clock in the evening when they came … there were 10-15 of them. Some had covered their face (…). They surrounded my house (…). When I went outside, they captured me all of a sudden and blindfolded me. Then they took me to a small river. They accused me of being CID [a spy]. They took me to the bank of the river and started beating me. They pointed the guns at me and acted as if they were going to kill me. They beat me very badly. I told them that I was a teacher at the village, not a spy, but they did not accept what I said (…). I told them that I teach students in the village. After that, they beat me a lot and in the end they broke my leg. They broke my leg into three pieces and left me (…). Two of them carried me and threw me on the front lawn of my house.

He went on to explain that it was since then that he felt his body trembling (*jieu thar thar garne*), felt weak, had nightmares, anticipated his attackers’ return as he lay half awake, and suffered from fear and heart ache—”It (the heart) doesn’t ache (physically), it’s not in my body”, he explained, “but it is in my *man* (heart–mind) and in my *dimāg* (brain–mind)”. Similarly, a farmer told us that he had suffered from fear and the anticipation of violence at nightfall since the time he was severely beaten until his aggressors thought he was unconscious.

Other participants considered that the onset of their symptoms was related to witnessing the killings and torture of others, their imprisonment, displacement and insecurity, and the many threats they had received throughout the time of the conflict. However, it is important to state that not all of study participants were plagued by physical or emotional symptoms. Time did appear to heal some wounds and moving forward was possible despite the memories, sadness, and grief. A woman who had lost her husband in the conflict and whose illness symptoms had become fewer summarized: “We have been surviving regardless of suffering or prosperity, with or without food.” Another participant reflected about how grief and social problems decreased over time saying, “There aren’t as many difficulties as there used to be before. There will always be problems, but you can’t make them into a mountain, you can’t think that this is how things are going to be, you can’t cry about it.” While time seemed to heal some wounds, these narratives also highlight that experiences of violence can have long-term impacts on people’s health and wellbeing; related memories continued to cause psychological and physical pain, fear, troubled sleep, and emotional heartache for many.

### The Interrelation Between War Experiences and Daily Stressors: Impact on Mental and Psychosocial Health

All study participants explained that their health problems were not solely caused or amplified by violent experiences and memories, but also by chronic, daily stressors. Unlike war experiences, daily stressors were mostly not particularly eventful or spectacular, but instead formed part of the daily routine of living, pertaining to participants’ economic, social, and familial situations. Yet, despite their structural differences, chronic daily stressors and the effects of the armed conflict were not experienced as separate from one another– they came together in people’s lives and, thereby, co-created illness experiences.

The Maoist Conflict had been fueled by people’s frustrations with economic inequality and poverty, and its outcome was perceived as a disappointment by many. All participants complained that their economic situation had not markedly improved due to the fact that subsistence farming did not yield enough income and available jobs did not pay enough to provide for children and purchase additional food and adequate clothing. A middle-aged widow stated:

I (often don’t) feel sleepy at night, and before I (know) it, it is light outside. I think about how I would raise my children. When they were little, there weren’t too many expenses for them, but the older they get, the more expenses they require. Look, my children are growing up, and they yell at me when I can’t fulfil their financial demands (…). My children also ask me what I am so worried about. My children tell me not worry, that we must find something or the other (a source of income). But, sometimes it’s like we can’t even eat dinner. Of course, I worry, why wouldn’t I worry.

Others complained that their dire economic situation did not allow them to pay for specialist doctors’ visits or medication. A middle-aged farmer who suffered from health problems related to the torture he had endured at the hands of government forces during the Maoist Conflict laid his situation out for us, explaining, “I don’t have any land of my own, I have to go plough others’ land. Fertilizer is expensive. If we don’t put fertilizer then there is no production, and when there is no production then I get worried about where I’m going to find the money for (medical) treatment”. Indeed, economic problems themselves were considered to cause or aggravate health problems and to impact on general wellbeing due to worries, sleeplessness, and problems with the heart-mind (*man*). Despite people’s overall negative perception of their economic situation, there were a few study participants (n = 4) who felt that their particular situation had improved due to the fact that their children were employed (n = 2) or that they received regular monetary support from family members who worked abroad (n = 2). With the extra help from family members, they were able build a new house, purchase some land for agricultural production, and support their basic needs.

Problems related to social status were also highlighted as negatively impacting on health and wellbeing. Particularly the narratives of war widows and persons with physical disability bring to the fore how war exposure and daily stressors intersect and co-create experiences of marginalization and ill health. All widows we talked to told us that the loss of their husbands during the armed conflict was devastating on multiple levels. Grieving for their husbands, they also had to face the consequences of losing their social status and respect among fellow villagers. A widow who continued to live with her in-laws in comfortable living conditions told us how radically her life had changed upon the loss of her husband, who was shot during a political campaign. “When I had my husband, many people used to come and chat … the life back then was so enjoyable. We used to be respected by so many. Many big people from the political party used to come here day and night.” After pausing to reflect she added, “Now, after he left us, I don’t know why, but no one comes to visit. It seems no one cares. People want to gather around burning wood. Who wants to gather around wood that is not burning?”

This sense of being considered cold and without purpose (wood that is not burning) led to exclusionary practices. This was illustrated by a widow who stated that others looked down upon her, related to her with disgust, and excluded her from social gathering and even family events. In frustration she exclaimed, “After our husbands die, we can’t die with them. And even though it is difficult, we have to carry on caring for our children.” Similarly, others complained that they might as well have died with their husbands instead of living a life at the margins of society, that others blamed them for the death of their husbands, and that they felt disabled without male support. “Well, without a husband around I feel limbless, I don’t have much support” a woman began. She continued, “When I walk around without a husband, I receive unfair treatment from others and (…) other people and my sons blame me.” Such statements were echoed by other widows who lacked support, had difficulty finding work with a decent income, and could not support their children financially while also protecting them from others’ prejudices.

The comparison between losing one’s husband and losing a limb was an apt one as men who suffered from torture-related physical disabilities also complained about the societal exclusion they experienced, disrespectful treatment and discriminatory practices which hindered them in finding employment. A middle-aged man whose leg had been broken and crushed by government forces felt that his disability had brought a part of his life to an end. He said, “Well, what can you do in life. If I were able to use my foot well, then I could go to someone else’s house and work. I would be able to live (from these earnings).” However, it was not just the missing limb, the disability, that kept men like him homebound. It was also the prejudice and lack of respect that people harbored towards the disabled. “I’m without a limb and they feel that whatever I do or say comes from a lowly level” someone said. Instead of being able to care for themselves, they perceived themselves as a burden for other family members who had to struggle to meet their and others’ needs. A former Maoist fighter, who was blinded as part of the torture that government prison guards had exposed him to, stated with resignation in his voice, “I must be fed, I must be cooked for, and going to the bathroom is whole other story…” His wife, a former Maoist fighter like her husband, struggled with “mental tension”, stomach pain, sleeping and eating problems, thinking too much and fatigue due to her memories of the violence she had been forced to endure and challenges connected to keeping the family farm running on her own. She was responsible for raising a small son, caring for her husband, keeping the house in order, and dealing with the cattle and agricultural production.

Strained family relations were an additional source of stress. While most participants considered their families supportive and generous, some women complained about being exploited and ill-treated by their in-laws. A widow shared with us that she had been used as a maidservant since her husband’s death while her in-laws remained idle and expected to be served. Consequently, she decided to leave their compound and live on her own despite struggling to secure an income and provide for her children. Two men, on the other hand, complained that their dreams and aspirations were cut short by their families who discouraged them from progressing in their education and careers. This, they stated, was an important source of frustration and stress. We were told, “They [my family] lack basic education, they don’t know anything (…) and can’t give advice. That’s why I don’t receive support from my family. I console my soul myself.” Others were distraught that their family members lived far away or even abroad and were, thus, unable to provide emotional support. The female Maoist fighter introduced above lamented, for instance, “Our family doesn’t live close by and I feel that if they did, then we could have talked and exchanged about the good and bad times we are experiencing.” Consequently, she often felt alone with her gruesome memories, emotional and physical pain, and pressure to provide for her husband and son.

One of the long-term effects of the Maoist Conflict was the intersection of traumatic memories with daily stressors. In their coming together, they were relentless and constant, affecting people’s social position through disability and widowhood; impacting on their ability to earn an income or perform daily tasks due to emotional and physical pain; and, as will become apparent in the following sections, affecting community cohesion, regional economy, and infrastructure.

### Coping and Help-Seeking

In order to ease the physical and emotional pain induced by traumatic memories and daily stressors, study participants resorted to various coping strategies including social activities, avoidance strategies and withdrawal, and substance use. Several explained that it helped them to find meaning in their illness through analogy, that is, by comparing their experiences to others (this corresponds to “salient prototypes” outlined in the MINI (56). They made their own symptoms more bearable by relativizing them in light of the suffering experienced by others. This, it was explained to us, made them realize that they were not alone with their pain and that others might have endured even more violence and loss. We were told, “When you have so much pain in your own mind, then you make yourself feel better by looking at the pain of others.” Similarly, a woman who had lost her daughter during the armed conflict explained:

Look, a lot of people have found themselves in this conflict. I think about the pain that other people have to go through and make myself feel better about my own situation. It is not only me who has lost something, (…) I tell myself some people have lost their husbands, some people have lost their wives, some have lost their daughters too, I am not the only one who has lost someone. I tell myself that everyone has pain and problems and that is how I console myself.

Although trauma and suffering were experienced at the level of the individual, the notion of shared grief and pain created a sense of companionship and solidarity which seemed to say ‘we are in this together’. Other times, companionship through social interaction was sought more actively as a form of distraction from one’s grief and worries and a means to escape loneliness and troubling thoughts. The same woman introduced above mentioned that since the death of her daughter, she could not stand being alone and consequently sought company by walking into the village to chat with her friends and visit acquaintances. Similarly, another woman told us, “During daytime, I talk to people while walking around and I don’t feel any pain. But when I am alone in the evening and start thinking, I experience such problems.” These examples make apparent that both illness and coping are social experiences which are shaped by historical and contemporary social change.

While illness and coping may be social, this does not mean that social relations are always sought after or that such relations are necessarily positive. We found that it was mostly men who tried to control their feelings by explicitly avoiding social company. They wandered around by themselves, visited temples or quiet places on their own, or worked harder than usual. For instance, a middle-aged man stated, “When I have the problem, then I keep working all the time. I feel pain, yet I keep working. But if I feel restless, and if it hinders my work, then I take medication. This is how I am living.” A few participants tried to relax or numb their feelings with alcohol or cigarettes. While alcohol was seen as a form of escapism, cigarettes were experienced as taking the tension away and were, accordingly, viewed as a form of relaxation by some. A woman who had lost both her son and daughter along with their friends had the urge to forget her sorrow. She exclaimed, “I couldn’t forget them and so I started drinking alcohol. I was so much hurt and I wanted to learn to smoke cigarettes. If I hadn’t learned smoking cigarettes, my headache would be severe. I was suffering badly so I learned how to smoke.” Alcohol-induced forgetting and pain relief for headaches through smoking felt like justified approaches, even skills (“I learned how to smoke”), to this woman in light of her suffering.

Help-seeking was mainly interrelational in that respondents requested and drew on family, community, and institutional supports. Most stated that it was their immediate family and friends who provided them with support. Family members provided material support in the form of money or financing the building or rebuilding of homes as well as social and emotional support and care. Similarly, friends and neighbors were perceived to be supportive in that they encouraged participants to socialize, listened to them, calmed them in times of distress, and/or tried to provide some comfort when in pain. A man with disability highlighted the importance of having forged a special type of bond (*mit lāune:* a tradition of friendship established through ritual) with a person suffering from similar health problems as their relationship provided him with a sense of solidarity and mutual understanding while, at the same time, it “freshened his mind” when he was feeling nervous or worried.

When asked about ways in which the village community provided support, participants provided examples of how, during the years of conflict, neighbors had protected each other during invasions, had taken children into their care whose families had been detained, or had driven the wounded to nearby clinics or hospitals. After the conflict, some communities had collected donations for the disabled in order to support them and their families as they were struggling to find a source of income. Only one person noted that a lack of communal solidarity and consequent feeling of isolation and frustration. He asked rhetorically, “What could the neighbors do? They are busy taking care of themselves. They are struggling to sustain their own families; how could they help us? We do have friends but who would be of help in times of trouble? Nobody will be there when you are in trouble, everyone supports you only in times of happiness. (…) I came to learn this.”

Most study participants had also received support from non-governmental organizations and governmental institutions in the immediate years following the conflict. The aid had been short-term and predominantly consisted of monetary and in-kind donations (i.e., food, clothes, building material) as well as psychosocial activities including self-help groups and trainings (i.e., training on livestock, agriculture, small business development, investment). While the aid was welcomed, it was often considered too short-term and insufficient. A widow highlighted that the government had provided her generously with 5 lakh (500,000 rupee) as seed capital but, from then on, only provided 150 rupee per month for her children– pocket money which she considered “not even enough to buy vegetables or a samosa.”

The major complaint was directed at the unfair distribution of aid which favored influential families, people with “connections” or those with ties to particular political parties. The resulting inequalities were experienced as stressful and as negatively impacting on people’s sense of wellbeing. A widow reflected on the unfair distribution of aid stating angrily, “Whoever has endured a painful conflict, that person will not receive any help. Whoever hasn’t endured conflict has received help from institutions. Even people who haven’t endured any conflict are making fake petitions and receive funding.” Similarly, others explained that there were those who wrote “fake claims” to receive funds based on their non-existent disability or veteran status or false description of their economic situation. We were also told about the distribution of funds based on political partisanship and nepotism as well as about politicians who attempted to increase their popularity and party membership by promising people access to welfare or in-kind donations. A man said, for instance, “(political) parties started to be biased saying ‘this victim is the one I know’ and started to provide (them with) much more relief and support (compared to) others.” Trying to verify these complaints, we interviewed a village leader who indeed acknowledged the study participants’ grievances as accurate, but, relativized their complaints by adding that many villagers were actually unaware of the eligibility criteria for welfare and aid, whom to approach to receive support, and what documentation to provide in order to file a claim. Nevertheless, he admitted that those working in administrative positions tend to ignore the situation and, thus, fail to engage in advocacy and knowledge transmission to improve access to welfare and other forms of support. Thus, aid was experienced as both a welcomed relief and a source of stress and communal conflict due to the way it was distributed, its scarcity and the lack of sustainability in a context where poverty and unemployment are widespread.

### Health-Seeking and Pathways to Care

Almost all study participants had received some form of biomedical treatment for their health problems as they visited a combination of doctors working in public and private sector clinics (n = 9); hospitals (n = 5) in Kathmandu, Palpa district, and India; mental health specialists (n = 2) including a neurologist and a psychiatrist; and professionals working for non-governmental organizations (n = 2) such as the ICRC or the Centre for Victims of Torture (CVICT). Although many received medication, most could not actually recall what kind, while some referred specifically to sleeping pills (n = 2), analgesics (n = 4), and medication for high blood pressure (n = 1). Medication was variously combined with other types of treatment and advice including scans (i.e. x-rays, CTs, MRI), psychiatric medication, counselling, advice from both doctors and family members not to worry or be preoccupied, exercise, and various forms of traditional healing.

Many felt that their health problems had improved over the years partly due to “time” and partly due to medical treatment, counselling and traditional healing. However, several participants felt that medication was not particularly effective as it was not perceived to lead to a cure. It was highlighted that medication had to be taken continuously while health problems kept re-surfacing at regular intervals despite the treatment. One man stated, “When I am alone, I think too much—if people are with me, then I think less. If I don’t take medicine then I can’t sleep at all. I can only sleep once I take the medicine that my son has sent me from India.” Another man explained that he had visited various doctors who had prescribed “four to five different medicines” which he considered ineffective. Finally, at a clinic in Gangalal he was prescribed medication he considered helpful and was willing to continue taking. Only one woman perceived the medication she had been prescribed to be completely unhelpful. She complained:

The pain from the death of my daughter caused so many types of illnesses. I spent about 12 lakhs (1.2 million) for my healthcare – the money that my eldest son had sent me was used to go to Kathmandu, Palpa and Lukhnow (India) where the doctors still couldn’t figure out what my problem was. (…) Even when I take the medicine that the hospital gave me, it hurts just as much as if I were not to take the medicine.

Interestingly only four participants said that they visited traditional healers for their health problems. We believe, however that this reflects under-reporting rather than actual health-seeking practice considering that well known healers lived in the area and existing literature highlights the widespread use of traditional healing for trauma-related as well as physical health problems. Those who reported seeking traditional healing visited both healers and temples to find inner peace and pain relief. For instance, we were told by one of our male participants that his illness had been variously interpreted as witchcraft by a *guruba* (traditional) healer and as neuroinflammation by a biomedical practitioner. When asked how he made sense of these different illness explanations, he responded:

A: (smiling) Who is correct? Both couldn’t do anything for me. Although the traditional healer couldn’t do anything, he did something. (…) I might consult others (traditional healers).

Q: So you think that you have been bewitched?

A: Yes, something like that.

Q: What did the *guruba* do to make you feel better?

A: He told me that using the traditional way through rituals (*jhaar phuuk*) would be correct.

The interviews made apparent that both biomedicine and traditional healing provided some relief, but failed to yield a lasting cure due to the chronic nature of the health problems, recurring traumatic memories, and ongoing socioeconomic stressors which were linked to psychological and physical pain. It also became apparent that there was no long-term mental health and psychosocial support program in place (most had travelled to other cities including Kathmandu to receive psychiatric treatment), nor did there appear to be any social interventions in the region that could directly address social and economic determinants of health and thus indirectly affect conflict-related memories and associated emotional pain.

## Study Limitations

This study has several limitations. First, the study was cross-sectional rather than longitudinal and based entirely on retrospective self-reporting of war-exposure and related illness symptoms. Although respondents indicated that their mental health problems originated from traumatic experiences endured during the Maoist Conflict, the actual time of onset of symptoms is impossible to determine. In fact, we suspect that most people experienced a number of triggers including conflict-related violence and structural violence as well as daily stressors experienced in the aftermath. Second, the study was based on a small sample and sampling was conducted in only one out of 77 districts in Nepal. Therefore, the results may not be representative of conflict-affected populations in other geographical areas or across all linguistic, caste, ethnic, and socioeconomic groups. Third, we failed to systematically collect information about ethnicity and participants’ socioeconomic status before the conflict. Consequently, it is impossible for us to more objectively determine whether or not respondents’ socioeconomic situation had changed due to or since the conflict. Despite these limitations, the data offer crucial insights into the long-term consequences of war and the ways in which people perceive war exposure and daily stressors to affect their mental health and wellbeing.

## Discussion and Conclusion

Our findings provide insight into the ways long-term consequences of war intersect with daily stressors and, in combination, affect people’s mental health and wellbeing. The findings further highlight the importance of designing interventions and support mechanisms that address not only immediate humanitarian, health and mental health needs, but also the social determinants of health (i.e., infrastructure, poverty, housing, education, and multiple forms of discrimination) which affect the context in which people are born, grow, live, work, and age (see 60). Respondents themselves highlighted how their illness experiences were co-produced by traumatic conflict-related experiences, extant social role and status-based discrimination (i.e., gender, widowed, unemployed, disabled), and daily stressors (i.e., insecure economic situation, impoverished living conditions, and lack of community and family cohesion). In combination, these various stressors were perceived to cause a wide range of symptoms spanning psychological, psychosomatic and organic domains. This finding corresponds with previous research in Nepal which has identified that people express distress through a vast number and range of symptoms (e.g., 18, 19, 35, 61; see Chase etal., (55) for a review of studies on culture and mental health in Nepal).

The onset of these symptoms was usually attributed to violence experienced during the Maoist Conflict. We were told about the gruesome events respondents had experienced and witnessed, the ways in which they relived these experiences through memories and dreams, and how they impacted them emotionally and physically even seven years later. Previous studies conducted in Nepal suggested that experiences of trauma was considered by survivors a consequence of their *karma* and fate (i.e., bad *karma* in the past leads to a bad fate) and hence stigmatization. Therefore, people preferred not to share their traumatic experiences (e.g., 37). However, we encountered no such attribution or any difficulty in talking about traumatic experiences (even experiences of sexual abuse and marital-rape were presented as index traumatic experience by the female survivors during trauma reactivation as part of the clinical trial).

Our research furthermore made apparent that while most participants attributed the onset of their symptoms to the armed conflict, they considered their overall illness experience to be co-produced by chronic daily stressors. The notion of co-production is in line with emerging evidence which suggests that social, economic, and physical environments in which people live shape their mental health (2, 16). Particular risk factors are poverty, social marginalization, isolation, inadequate housing, and changes in family structure and functioning (15, 62) (for Nepal see 29, 30, 63). Additionally, research has revealed that experiencing mental illness increases the risk for poor social conditions and weak social ties (15, 64).

We would like to further stress the notion of “co-production” as it also informed people’s coping, help- and health-seeking behaviors, as well as reflections on social support and medical treatment. Similar insight is provided by Thapa and Hauff (30) who found that perceived needs among displaced people during the Maoist Conflict included financial needs, housing, food, and education for their children. The authors conclude that responding to “perceived needs of job opportunities and social help (…) could potentially address mental health problems” (p. 593). However, in both their and our study, such support was hard to come by due to the inadequate healthcare and social welfare system and lacking resources and infrastructure.

To address mental health problems experienced by survivors of war as well as the general population in the long-term, we advocate for a multi-sectoral social, medical, and psychological approach that pays attention, first and foremost, to the political, social and economic determinants of health. There is growing evidence on such intersectoral interventions to alleviate the effects of poverty, inequality, and social marginalization on mental health by addressing upstream factors (15, 16). Downstream, specialized psychiatric support should be reserved for people suffering from trauma-related health problems such as PTSD and clinical depression (65). Having said this, we are well aware that it has proven challenging to find the right place for upstream interventions since potential targets extend well beyond mental health services to include the individual, family, community and social, as well as political levels (15, 16, 66). A further complicating factor is that interventions should not only be intersectoral but also context-specific, requiring a good understanding of the respective local history and political, socioeconomic, cultural situation.

To illustrate the different intervention levels that could address mental health problems and distress in the context of our research, we have adapted a table produced by Wahlbeck et al. [(16), p. 506]. (See **Table 2**).

**Table 2:** Levels and targets of interventions to improve mental health of survivors of war in Dang district, Nepal (Adapted from 16).

***Country level***
Reduction of poverty and inequality with attention to caste-ethnic and religious affiliation, gender, and urban/rural divides; reduction of discrimination with attention to particularly marginalized groups (e.g., widows and people with disabilities); improvement of infrastructure and road safety which would allow people to access available health and social services; improvement of governance; protection of human rights; development and implementation of national policies to ensure access to education, employment, decent wages, health care, and housing proportionate to need irrespective of social status.
***Service level***
Accessibility and improvement of schools and adolescent/youth services; provision of health care with attention to existing pluralistic medical traditions (i.e. Ayurveda, Tibetan medicine, ritual or faith-based healing, astrology, herbal medicine, homeopathy and biomedicine); provision of specialized services for survivors of torture and gender-based violence; development and delivery of social and welfare services; creation of a referral system connecting medical, psychosocial and social services; provision of clean water and sanitation.
***Community level***
Creation of an information system enabling access to health and social services; building of neighbourhood trust/cohesion and safety; carrying out community-needs assessments; respect for places for spiritual/religious healing and communal practice/worship/celebration; enabling of community involvement and participation with attention to local power dynamics related to socioeconomic status, caste-ethnic and religious affiliation, age, gender, and disability; creation and implementation of support groups for survivors of violence and loss; prevention of violence/crime; reduction of social and economic deprivation at the neighbourhood level.
***Household and working life level***
Support for families and strengthening support within and across extended families with attention to loss of family members during the conflict and change in social position of female members, esp. daughters-in-law and widows; improvement of material conditions (i.e., income, access to resources to fulfil basic needs); improvement of employment conditions and decrease in unemployment (with attention to agriculture); provision of decedent wages; provision of pregnancy and maternal health care and social support.
***Life course level***
Health and social support provision during prenatal, pregnancy and perinatal periods; early childhood; adolescence; working and family building years; and older age, all with attention to social position and gender.

Such a multi-level inter-sectoral approach would require a bringing together of so far fragmented services and spheres (67). Achieving this would not only have the potential to improve mental health and wellbeing, but would also address other concerns which form part of the Sustainable Development Goals. Mental health is now explicitly mentioned as part of SDG 3, highlighting the inclusion of mental health care in universal health coverage. This mentioning is important; yet, it does not account for the fact that mental health treatment alone will have limited capability to enhance mental health and wellbeing considering that research has shown that mental disorders are strongly socially, economically and politically determined (68). These determinants, in turn, can be linked to various SDGs as they relate to poverty, nutrition, education, gender and other social inequalities, living conditions, decent work, and strong institutions. Thus, in order to address mental ill health, it is crucial to work across sectors and paying attention to the “multidimensional way in which [political, social and economic] determinants interact with key genetic determinants to affect mental disorders” (68, p. 357). In order to achieve this, it will be hugely important to foster the participation of and partnerships between government sectors, civil society organizations, and affected people and their communities. Only through such a holistic and context-specific approach will it be possible to provide populations, including survivors of war, with adequate healthcare, a secure environment, and the ability to live lives they and their communities value.

## Data Availability Statement

The datasets generated for this study are available on request to the corresponding author.

## Ethics Statement

This study was carried out in accordance with the recommendations of the Research Ethics Board of the Douglas Mental Health University Institute in Montreal with written informed consent from all subjects. All subjects gave written informed consent in accordance with the Declaration of Helsinki. The protocol was approved by the the Research Ethics Board of the Douglas Mental Health University Institute in Montreal, Canada and from the *ad hoc* ethical committee at the Department of Psychiatry and Mental Health at Tribhuvan University Teaching Hospital in Kathmandu, Nepal. 

## Author Contributions

HK is the Principal Investigator of the project, developed the research tools, conducted half of the interviews, coded and analyzed the data, and wrote most of the article. RP worked as a research assistant on the grant, developed the research tools, conducted half of the interviews, coded and analyzed the data, contributed to the writing, and provided feedback. 

## Funding

Funding for this project was received from Grand Challenges Canada. This publication was also funded through the UK Research and Innovation GCRF RESEARCH FOR HEALTH IN CONFLICT (R4HC-MENA); developing capability, partnerships and research in the Middle and Near East (MENA) ES/P010962/1. The funder contributed to the analysis, writing and dissemination of our work. 

## Conflict of Interest

The authors declare that the research was conducted in the absence of any commercial or financial relationships that could be construed as a potential conflict of interest.

The handling editor declared a past co-authorship with the author HK.

## Footnotes

1 According to the MINI, prototypes “involve reasoning based upon salient episodes or events in one’s own or others’ experiences, which allow individuals to elaborate the meaning of their illness through analogy (Example: ‘Last year, my uncle and aunt died of lung cancer, so I got scared and decided to quit smoking’)” (Groleau, D., A. Young and L.J. Kirmayer. 2006. The McGill illness narrative interview (MINI): An interview schedule to elicit meanings and modes of reasoning related to illness experience. Transcultural Psychiatry 43, no 4: 671–91).

2
*Aurāhā/audāhā* (feeling hot and suddenly angry), *Bhalbhal hune* (boiling sensation), Bad thoughts, Boredom, Breathing problems, Burning sensation, Confusion, Current in the body (feels like it), Dark mind, Depressed, Digestion problems, Dull bodily feeling, Dysentery, Fainting, Feeling allergic toward people, Feeling cold while it was warm outside, Feeling empty, Gastritis, Hearing voices, Heart stops beating (feels like it), Helplessness, Hopeless, Inability to do something, Isolation, Loneliness, Loss of control, Nerves feel like breaking, Nervousness, Panicked body, Rib pain, Sinking feeling, Stammering, Suicidal ideation, Swelling of hands, Swelling of joints, Tired, Trembling, Uncommunicative, Visions, Vomiting.
